# Challenges of scaling up cervical cancer screening in Ethiopia: a qualitative socio-ecological study

**DOI:** 10.1016/j.eclinm.2026.104057

**Published:** 2026-07-09

**Authors:** Begetayinoral Kussia Lahole, Abayneh Tunje Tanga, Firehiwot Haile Abdi, Selamawit Fisseha Mekuria

**Affiliations:** a Department of Midwifery, College of Medicine and Health Sciences, Arba Minch University, Arba Minch, Ethiopia; b School of Public Health, College of Medicine and Health Sciences, Arba Minch University, Arba Minch, Ethiopia; c Department of Medical Oncology, Faculty of Medicine, Lund University, Lund, Sweden

**Keywords:** Cervical cancer screening, Qualitative research, Social ecological model, Ethiopia

## Abstract

**Background:**

Cervical cancer remains the leading cause of cancer-related death among Ethiopian women, second only to breast cancer. Previous qualitative studies have simply listed the barriers and facilitators without showing how the identified barriers interact across different levels to result in low screening coverage despite national efforts. Guided by the socio-ecological model (SEM), we aimed to explore barriers and facilitators influencing cervical cancer screening uptake in Ethiopia focusing on the dynamic interplay between women, their social networks, the health system, the community, and the policy at large.

**Methods:**

A descriptive qualitative study design was employed among women, male partners, community and religious leaders, service providers, and health officials. The study was guided by the previously published SEM. Focused group discussions (FGDs), in-depth interviews (IDIs), and key informant interviews (KIIs) were conducted, and the audio recordings were transcribed, translated, and analyzed using Braun and Clarke's reflexive thematic analysis, supported by ATLAS.ti version nine software. The entire research team was involved in several rounds of discussion and consensus-building to refine themes and sub-themes. The study was conducted in four zones of the South Ethiopia Region in June 2025.

**Findings:**

A total of 104 participants were included: 56 women in seven FGDs, 34 in KIIs, and 14 in IDIs. Barriers and facilitators did not act in isolation but interacted dynamically across SEM levels. At the individual level, fear of procedure and symptom-based screening were reinforced by interpersonal barriers, particularly fear of divorce following a positive diagnosis. Institutionally, service integration facilitates, but supply failures and transport costs erode trust. Community-level religious leaders endorse screening, yet prayer-based beliefs override this. Policy-level partnerships enable programs, but weak funding and broken equipment undermine delivery. Policy achievements do not translate into reliable frontline services.

**Interpretation:**

According to the findings of this study, the complex interaction of barriers and facilitators across all levels of the SEM underscores the importance of multi-level implementation strategies to achieving treatment follow-up uptake.

**Funding:**

Mrs Berta Kamprad Cancer research FBKS-2022-24–(432).

**Research in context**

**Evidence before this study**

We searched PubMed and Google Scholar from January 1, 2000, to December 30, 2025, for papers published in English, using the terms “cervical cancer”, “cervical screening”, “Ethiopia”, “qualitative”, “focus group”, and “interview.” Our search yielded 151 results. Many quantitative studies conducted in Ethiopia highlighted a low uptake of cervical cancer screening and the factors associated with it, and very few qualitative studies have explored the individual and institution-level challenges contributing to such a low level of utilization. However, no comprehensive qualitative evidence exists on screening challenges involving the individual, interpersonal, community, institutional, and policy levels to inform policy and direct future cervical cancer implementation projects.

**Added value of this study**

Guided by the SEM, this qualitative study is the first to explore the “why” behind women's decisions to be screened or not screened from multiple perspectives. Our study revealed how a cycle of mistrust is created by the failed health system, which directly undermines individuals' self-efficacy and community mobilization efforts. Notably, after exploring the perceptions and lived experiences of women, husbands, community leaders, religious leaders, service providers, and higher health officials, this study uncovered why the ongoing efforts in Ethiopia failed to achieve high screening uptake. Therefore, the findings of this study will inform implementation scientists in designing effective and multi-level intervention strategies in the country.

**Implications of all the available evidence**

To successfully increase the uptake of cervical cancer screening in Ethiopia, a multifaceted intervention should be used. Cervical cancer should be integrated into current maternal health units, barriers at all levels should be addressed, and an uninterrupted supply of screening resources should strengthen the failing health system. In addition, involving community gatekeepers and the women's development army is essential to narrowing the gaps and dismantling stigma. Strong leadership commitment and strengthening external partnerships are needed at the policy level.

## Introduction

Despite being largely preventable, cervical cancer remains a leading cause of cancer-related mortality in low- and middle-income countries (LMICs), with sub-Saharan Africa carrying the highest disease burden.^1^^,^^2^ In Ethiopia, 8168 new cases and 5975 deaths were documented in 2023, and without effective intervention, projected cervical cancer deaths are estimated to reach 191, 876 by 2070 and 275, 990 by 2120.^3^^,^^4^

In response to this growing burden, in 2020, the World Health Organization (WHO) set a triple intervention pillar to be achieved by 2030 that includes 90% vaccine coverage, 70% screening uptake, and 90% treatment and care of detected cases.^5^ However, although Ethiopia has made efforts to expand cervical cancer screening through national strategies such as the 2015 National Cancer Control Plan and the 2016 “screen-and-treat” approach using visual inspection with acetic acid (VIA) and cryotherapy,^3^^,^^6^^,^^7^ coverage remained very low. The Ethiopian Federal Ministry of Health led these efforts in collaboration with partners like the WHO, but coverage was only about 3% in 2020, far below the WHO target.^4^ The South Ethiopia region, where the current study was conducted, follows a same-day VIA “screen-and-treat” approach similar to other parts of Ethiopia. This screening is provided by midwives, nurses, and physicians free of charge for women aged 30–49 at health centers and hospitals. Despite the screening service being available in the region, only 12·6% of eligible women had ever been screened for cervical cancer as of 2020.^8^

Even if several quantitative studies have identified factors associated with low screening uptake,^9^, ^10^, ^11^, ^12^, ^13^ and a few qualitative studies have explored barriers and facilitators at the individual or institutional level,^14^^,^^15^ multi-level qualitative evidence to inform implementation and policy remains limited. Moreover, the limitation of previous qualitative studies is that they simply listed the barriers and facilitators without showing how the identified barriers interact across different levels to result in low screening coverage despite national efforts. This study, employing a multi-stakeholder qualitative approach, therefore, examines how the barriers and facilitators of cervical cancer screening interact across the individual, interpersonal, community, institutional, and policy levels in South Ethiopia.

The SEM, specifically McLeroy's ecological framework,^16^ was used to guide this study. The reason we chose the SEM instead of other models, such as the Consolidated Framework for Implementation Research (CFIR) or Reach, Effectiveness, Adoption, Implementation, and Maintenance (RE-AIM), is that the aim of our research was to understand women's lived experiences and the interplay of barriers across levels, rather than to evaluate intervention fidelity or scalability. Moreover, the reason we specifically chose McLeroy's framework from other available ecological models was that it focuses on how individuals navigate and are influenced by proximal processes across interconnected systems over time, which is particularly suited for understanding health behavior decision-making. It comprises five integrated levels (Supplementary Material S1): individual, interpersonal, community, organizational, and policy. Using this framework, the study aims to provide a comprehensive understanding of the key facilitators and barriers to cervical cancer screening at various levels and how they interact across levels. Furthermore, this study aims to inform policies and provide knowledge for future strategies dealing with the integration of Human Papillomavirus (HPV)-based cervical cancer screening into existing health platforms in Ethiopia.

## Methods

### Study design and setting

**Fig. 1:**
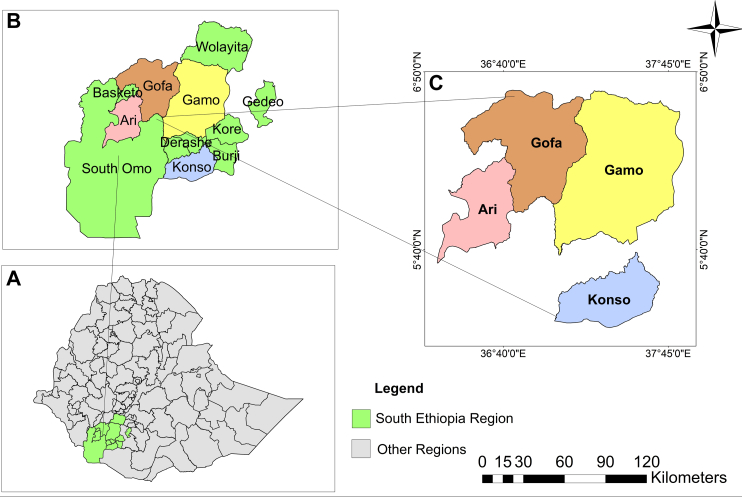
**Map of the study area, South Ethiopia Region.** Notes: (A) Location of South Ethiopia Region within Ethiopia. (B) South Ethiopia Region and its 12 zones. (C) The four zones included in the study.

A qualitative descriptive design^17^ was employed as it is well-suited to address the research questions therefore allowing for a concise and clear exploration of the beliefs, views, and lived experiences of women, male partners, community leaders, religious leaders, healthcare professionals, and health officials in their own words in Gamo, Gofa, Konso, and Ari Zones of the South Ethiopia Region in June 2025 (Fig. 1). The region comprises 12 Zones and 70 Districts with 41 hospitals, 300 health centers, and 1500 health posts.^18^ The estimated total population of the region is 4·5 million, of whom 2·3 million are of reproductive age women. The region has an approximated 52% of female literacy rate with significant variation across zones (38% in Konso Zone to 68% in Gamo Zone). The population is predominantly rural, with approximately 85% of residents living in rural areas and relying on subsistence agriculture.^19^ The four Zones included in the current study were purposively selected to capture variation in geographic, cultural, and health system characteristics to enhance the transferability of findings across similar settings in Ethiopia. Gamo Zone is characterized by mostly highland areas, with Arba Minch City acting as an important referral center. Gofa Zone includes both lowland and highland areas with little access to roads. Konso Zone is characterized by semi-arid lowlands. The Ari Zone comprises lowland areas bordering the Omo River basin. Within this region, a total of 47 health facilities provide cervical cancer screening and treatment services, including 32 health centers, 12 primary hospitals, and four general hospitals.^18^ Integration of cervical cancer screening with family planning and antiretroviral therapy (ART) services was present only in Ari and Gofa zones at the time of data collection; Gamo and Konso zones did not have established integration.

### Participant's recruitment

A purposive sampling strategy was used to capture diverse sociocultural perspectives on facilitators and barriers to cervical cancer screening. Participants included screened and unscreened women; women living with HIV (WLHIV); women on, lost to, or having completed treatment follow-up; health professionals from different units; health bureau officials; husbands; community leaders; and religious leaders. Health extension workers (HEWs) assisted in recruiting screened and unscreened women from the communities. With the help of HEWs, the physical address of women who were lost to follow-up was identified from the screening logbook and located in the community. Despite these efforts, only two of such women were identified and interviewed. Although recruitment from this subgroup was limited, the initial target of approximately 100 participants across all groups was met (final N = 104). Fewer participants were recruited from Konso Zone than from the other study zones because cervical cancer screening services were less established and less frequently utilized during the study period, resulting in fewer eligible women being identified for recruitment. Women were divided into distinct FGDs based on their screening status to avoid social desirability bias and allow unscreened women to freely share barriers. Each FGD included women in a 10-year age span (30–39 or 40–49) to guarantee age homogeneity and participant comfort.

### Data collection

FGDs, IDIs, and KIIs were conducted face to face by experienced and trained qualitative data collectors (BKL and FHA) who are proficient in the local language and experienced in discussing sensitive health topics. The FGD and interview guides (Supplementary Material S2) were developed based on the five SEM levels and informed by prior literature on cervical cancer screening barriers in LMICs^9^, ^10^, ^11^, ^12^, ^13^ and piloted with four participants and refined based on the findings of the pilot study to enhance relevance and clarity. All FGDs and interviews were conducted in the Amharic language. Interviews and FGDs took place in private, quiet, and accessible settings to ensure confidentiality and data quality. We conducted seven FGDs, separately with screened and unscreened women, to explore facilitators, barriers, shared norms, and group-level perceptions of cervical cancer screening. Each FGD was facilitated by a team of three (moderator, note-taker, and, in the Ari Zone, a translator to provide occasional clarification of questions in the local language, as some participants expressed themselves more comfortably in their mother tongue while fully understanding Amharic), included 8–12 participants, and lasted 60–90 min. Additionally, 14 in-depth interviews were completed with WLHIV, women lost to follow-up, women currently on follow-up, community gatekeepers (religious and community leaders), and male partners to capture in-depth beliefs and personal experiences. A total of thirty-four KIIs were conducted with service providers working in different units and health bureau officials to gather detailed information at the service delivery and policy levels. Both IDIs and KIIs lasted an average of 45 min and were conducted by experienced qualitative data collectors. Highlights during the data collection were recorded in a notebook. Transcription and translation of the recordings from the local language into English were subsequently made for analysis. Translation accuracy was validated via back-translation of a random 20% of transcripts by an independent bilingual translator. Agreement on key concepts was made and discrepancies were resolved through discussion among the research team. Thematic saturation was pursued through iterative data collection and analysis. Recruitment continued until no new substantive themes emerged from the data. Specifically, thematic saturation for the knowledge gap theme was reached after four FGDs, with no new insights emerging from subsequent groups.

### Data analysis

The McLeroy's SEM^16^ was used to guide this study, which emphasizes nested, dynamic interactions between individuals and their environment across multiple levels. The six steps of Braun and Clarke's reflexive thematic analysis^20^ were followed to analyze our data, which included 1) Data familiarization, 2) Initial code generation, 3) Identifying initial themes, 4) Reviewing themes, 5) Defining and naming themes, and 6) writing a report. Data transcription and translation were primarily made by Begetayinoral Kussia Lahole (BKL) and Firehiwot Haile Abdi (FHA). Before data analysis, each recorded audio and transcribed data was cross-checked to identify and/or include any additional overlooked ideas. Senior researchers in the team Selamawit Fisseha Mekuria (SFM) and Abayneh Tunje Tanga (ATT) and other researchers from cross-disciplinary backgrounds were consulted to ensure trustworthiness and accuracy of our transcriptions and translations. This process of data familiarization and verification created a deep immersion with the data before systematic coding was started. No formal distinction was made in the analytical handling of FGDs, IDIs, and KIIs; all transcripts were coded together using the same inductive process. However, comparisons were systematically made across participant groups to identify group-specific themes. ATLAS.ti 9 qualitative data analysis software was used to analyze the data. BKL conducted the initial coding of all transcripts, and an inductive coding scheme was developed from the initial codes through iterative discussions conducted with the co-authors. These were further developed into sub-themes and themes (Supplementary Material S3). Although a formal Delphi method was not used, the entire research team was involved in three rounds of discussion conducted over two weeks to refine the sub-themes and themes. This study followed the Consolidated Criteria for Reporting Qualitative Research (COREQ) guidelines.^21^ The completed COREQ checklist is provided as a Supplementary File.

### Researcher's characteristics and reflexivity

This qualitative study builds upon SFM's PhD thesis, “Towards improved cervical cancer screening in Ethiopia.” Her PhD involved the evaluation of HPV-related algorithms for LMICs, including HPV self-sampling. SFM is a postdoctoral fellow in medical oncology at Lund University in Sweden and an obstetrician and gynecologist with a wealth of clinical expertise. BKL, a male researcher, holds a Master of Science in Clinical Midwifery and has clinical and research experience, which allowed him to engage deeply with study participants and create a professional and empathetic environment during the data collection process. FHA, a female researcher, has a clinical nursing background and a Master of Public Health in Reproductive Health, which has been valuable both for data collection and data analysis. BKL and FHA introduced themselves to study participants and explained the purpose of the study before obtaining consent. ATT, an epidemiologist, has a wealth of experience in public health research and in managing large-scale research projects, which was very helpful to the research team. Except for SFM, who is based in Sweden, all researchers are Ethiopian and reside in the study region. This provided unique access and cultural familiarity but also introduced potential assumptions about local beliefs and practices. SFM's training as a gynecologist in Sweden may have introduced a Western medical perspective, while ATT's epidemiologic background may have inclined the team toward quantitative reasoning.

Although BKL's participation in FGDs with screened and unscreened women as a male midwife allowed him to engage directly with the participants, it may have introduced discomfort and bias. However, male midwives are now often found in Ethiopian healthcare institutions, and most gynecologists in the country are male. Women in Ethiopia are therefore not new to discussing sensitive reproductive health matters with male providers. Moreover, to reduce potential bias, first, participants were told they could choose between BKL and FHA prior to data collection. Second, FHA took the lead on sensitive topics during FGDs and conducted some interviews based on the preferences of the interviewees. Third, all team members engaged in ongoing reflexivity through regular discussions about how our backgrounds might shape data collection and interpretation.

### Ethics

Ethical clearance was obtained from the Institutional Research Ethics Review Board (IRB) of Arba Minch University (Reference Number: IRB/23337/25). The study adhered to the principles of the Declaration of Helsinki. Before the actual data collection started, written informed consent was obtained from all study participants, including consent for audio-recording and publication of anonymized quotes. Moreover, participants were told their rights to withdraw from the study and to skip questions they don't want to answer, at any time, without negative consequences. Confidentiality was ensured through anonymisation and secured data handling.

### Role of the funding source

The funder of the study had no role in study design, data collection, data analysis, data interpretation, or writing of the report.

## Results

### Characteristics of study participants

A total of 104 individuals participated in this study, with 56 women included in seven FGDs, 34 participants taking part in KIIs, and 14 participants engaged in IDIs. The FGDs were conducted across all Zones comprising women aged 30–49 years. The KIIs comprise health professionals working at various units and health bureau officials. The IDIs included religious and community leaders, husbands, and women. Among male participants (n = 26), husbands comprised the largest subgroup (n = 12, 46·2%), while community leaders formed the smallest subgroup (n = 4, 15·4%) (Table 1).

**Table 1:** Socio-demographic and professional characteristics of study participants (N = 104).

Characteristic	Category	Total (N = 104), n (%)	Screened women (n = 34), n (%)	Unscreened women (n = 44), n (%)
Sex	Female	78 (75·0)	–	–
	Male	26 (25·0)	–	–
Male participants by role (n = 26)	Husbands of women	12 (46·2)	–	–
	Religious leaders	8 (30·8)	–	–
	Community leaders	4 (15·4)	–	–
	Health professionals	2 (7·7)	–	–
Age group (all participants)	18–29	30 (28·8)	–	–
	30–39	41 (39·4)	–	–
	40–49	22 (21·2)	–	–
	≥50	11 (10·6)	–	–
Age group (women only, n = 78)	18–29	8 (10·3)	0 (0·0)	8 (18·2)
	30–39	41 (52·6)	14 (41·2)	27 (61·4)
	40–49	22 (28·2)	20 (58·8)	2 (4·5)
	≥50	7 (9·0)	0 (0·0)	7 (15·9)
Participants per zone (all participants)	Gamo	30 (28·8)	–	–
	Gofa	30 (28·8)	–	–
	Ari	30 (28·8)	–	–
	Konso	14 (13·5)	–	–
Participants per zone (women only, n = 78)	Gamo	22 (28·2)	11 (32·4)	11 (25·0)
	Gofa	22 (28·2)	10 (29·4)	12 (27·3)
	Ari	22 (28·2)	9 (26·5)	13 (29·5)
	Konso	12 (15·4)	4 (11·8)	8 (18·2)
Educational status (all participants)	No formal education	22 (21·2)	–	–
	Primary education (1–8)	30 (28·8)	–	–
	Secondary education (9–12)	20 (19·2)	–	–
	College/University and above	32 (30·8)	–	–
Educational status (women only, n = 78)	No formal education	22 (28·2)	4 (11·8)	18 (40·9)
	Primary education (1–8)	24 (30·8)	8 (23·5)	16 (36·4)
	Secondary education (9–12)	15 (19·2)	9 (26·5)	6 (13·6)
	College/University and above	17 (21·8)	13 (38·2)	4 (9·1)
Participant category (all participants)	FGD participants	56 (53·8)	–	–
	Key informant interviews	34 (32·7)	–	–
	In-depth interviews	14 (13·5)	–	–
Professional role among KII participants (n = 34)	Midwife/Nurse (VIA, family planning (FP), antenatal care (ANC))	12 (35·3)	–	–
	Health bureau official	8 (23·5)	–	–
	Laboratory technician	5 (14·7)	–	–
	Health officer/manager	5 (14·7)	–	–
	Gynecologist/Medical doctor	4 (11·8)	–	–

Participants in the 18–29 and ≥ 50 age groups were exclusively from KIIs and IDIs.

Percentages for Professional Role are calculated out of total KII participants (n = 34).

### Themes and subthemes

Themes and sub-themes were categorized into the five interrelated constructs of the SEM (individual, interpersonal, community, organizational, and policy level), which enable us to capture the multi-level barriers and facilitators of cervical cancer screening from the individual to the policy level (Supplementary Material S3).

### Theme 1: individual level: knowledge, beliefs, and personal experiences

#### Facilitators at the individual level

The most influential facilitator at this level was strong motivation from personal exposure to cervical cancer, including knowing someone with the disease. This facilitator was reported by screened women across multiple FGDs, indicating it was not an isolated anecdote but a recurring theme. Provider counseling was also mentioned by many screened women as an important facilitator. Counseling about the severity of the disease, prevention, and treatment changed the narrative from fear of screening to women's empowerment.

“Previously, I was reluctant to go to the screening despite the information I received from the providers during my hospital visit for family planning service. The recent death of my sister from cervical cancer was the main reason for me to go to the screening,” (Screened woman, 32 years), in contrast to provider-initiated motivation. “The healthcare providers told me about how serious cervical cancer is, especially if it is not identified at an early stage. They also told me that if it is identified and treated early, it is curable. I decided to receive the screening after they told me this,” (Screened woman, 34 years).

#### Barriers at the individual level

Low health literacy, symptom-based screening behavior, fear of the screening procedure, and fatalistic beliefs were the key individual-level barriers. The most commonly reported barrier was limited health literacy, reflected in lack of awareness about cervical cancer and screening services. Many women also cited symptom-based screening behavior and fear of the procedure. Women who were lost to follow-up specifically cited fear of the procedure as a primary reason for discontinuing care. In contrast, fatalistic beliefs were mentioned by only a few women. For many women, feeling fine and having no symptoms were the main reasons that prevented them from going to the screening.

“I felt fine and had no symptoms, so I didn't see the need to get screened,” (Unscreened woman, 42 years), alongside limited awareness of screening services. “I just heard about it now, but I didn’t know before,” (Unscreened woman, 33 years).

### Theme 2: interpersonal dynamics: the role of family, partners, and healthcare providers

#### Facilitators at the interpersonal level

Support from the women's social environment, such as husbands, peers, and healthcare providers, positively influenced the screening uptake. Among these, peer experience sharing was more commonly reported than husband support. The encouragement from supportive spouses has resulted in screening and treatment adherence, while a respectful approach from providers and peer testimonials further strengthened the women's screening participation. “When a wife gets an illness, it's her husband's illness as well. He will be affected by her pain. Hence, husbands have a huge role in cervical cancer screening,” (Husband, 41 years), while peers contributed through shared experiences: “When we share with our neighbors, it's not scary; we have experienced it ourselves, and women who were hesitant about the screening will feel encouraged when they listen to our stories” (Screened woman, 31 years).

#### Barriers at the interpersonal level

On the contrary, the same social environment can affect women's intention to screen. Fears of divorce and partner opposition were more commonly reported barriers than lack of partner permission, indicating that men more often impeded rather than enabled screening participation in this setting. For some women who become worried that a diagnosis could lead to divorce, the adverse reaction from their husbands remained the major concern and source of fear.

“If I get screened and they tell me I have cervical cancer, my husband will leave me. He will find another wife. He needs a healthy woman. Then I will be a woman whose husband left her because of the disease. So I am afraid to get the screening,” (Unscreened woman, 31 years).

Notably, a contradiction was observed between husbands' perspectives and women's lived experiences. Although some husbands described themselves as supportive, many women avoided screening due to fear of divorce and partner opposition. This reflects underlying gender dynamics that shape screening decisions, where perceived partner support does not necessarily translate into women's lived realities.

### Theme 3: institutional context: healthcare system facilitators and barriers

#### Facilitators at the institutional level

Screening uptake was made easier by the integration of cervical cancer into already-existing maternal health units, such as family planning, ART, and outpatient departments, along with organized monitoring and referral follow-up. This was the most commonly cited facilitator at the institutional level. WLHIV reported higher screening uptake specifically due to this integration of cervical cancer screening with ART services.

“In our hospital, what makes the uptake of cervical cancer screening high compared to hospitals in other Zones is the established systematic referral linkage of women who initially come for other reasons. Women will be referred from outpatient departments, family planning units, and ART clinics to the VIA clinic,” (Service provider, 32 years).

#### Barriers at the institutional level

Geographical inaccessibility, high transportation costs, lengthy wait times, insufficient privacy, a lack of supplies, and broken treatment equipment were the primary obstacles at this level. The most frequently mentioned of them were supply shortages and treatment machine failures, followed by transportation costs and geographic inaccessibility. Treatment delays, service interruptions, and a drop in public trust in the healthcare system were the results of these challenges. WLHIV, despite higher initial screening uptake, faced similar barriers to follow-up care as the general population, including supply shortages and transportation costs. Women lost to follow-up identified transport costs as a primary reason for discontinuing care, consistent with the experiences of unscreened women.

“The health facility is very remote. Travel costs by motorcycle and car are 200 birr∗ and 70 birr, respectively. We can’t afford the travel cost,” (Unscreened woman, 41 years), whereas the high cost and prolonged time taken for refilling the CO_2_ cylinders required for cryotherapy remained the main challenge “refilling the cylinders is too costly since it requires traveling to Addis Ababa. This supply chain failure directly affects the screening service and the patient's experience, as it delays the treatment,” (Health official, 38 years).

Importantly, a divergence was observed in how these institutional challenges were understood across stakeholder groups. While providers and health officials framed these issues primarily as system-level constraints, women often interpreted repeated service interruptions and delays as a lack of health system reliability. This perception appeared to reduce trust in the screening service and influence their willingness to seek care.

∗ETB (Ethiopian birr); 200 ETB was approximately equivalent to US$1·50 and 70 ETB to US$0·50 at the time of the study.

### Theme 4: community and cultural environment

#### Facilitators at the community level

Religious and community leaders were influential facilitators of screening uptake when engaged as partners.This was mentioned by most religious leaders interviewed. Their involvement enabled large-scale community mobilization and improved trust in screening initiatives.

“If you educate us about cervical cancer and its prevention first, we will utilize organized instruction to mobilize the community. Thousands of women can be reached in a single Sunday service,” (Religious leader, 58 years).

#### Barriers at the community level

Misinformation about cervical cancer, limited awareness, and wrong religious beliefs about healthcare utilization remained the key barriers at this stage. Limited community awareness was universally reported by community leaders, while religious beliefs discouraging screening were mentioned by approximately half of unscreened women. Thus, while religious leaders themselves were willing facilitators when engaged, religious beliefs held by some community members served as a barrier. Some religious teachings promoted prayer-based healing over medical treatment, reinforcing fear and mistrust in the community.

“The community's awareness is very low. They don’t understand why the screening is needed, why early detection is important. There is a significant knowledge gap in our community,” (Community leader, 51 years), particularly where religious beliefs discouraged modern healthcare: “Some extremist or newly emerging religions claim modern health technologies are ‘666’ and discourage screening. This misleads people,” (Community leader, 49 years).

“666” is a symbolic reference used in some religious interpretations to denote evil or anti-religious practices.

### Theme 5: the role of public policy

#### Facilitators at the policy level

Working with external partners and the integration of cervical cancer screening into the HIV/AIDS program were the main facilitators identified at this level. These were mentioned by all health officials interviewed. It is well understood that the entire screening program relies on policies and resources. If the policies are weak, many efforts at other levels will be difficult to maintain.

“The role of external partners was very important to maximize the screening uptake. If the screening solely relies on the public system, a chronic shortage of consumable supplies and treatment machines will result in service interruption and even stoppage. Moreover, I am working under the HIV/AIDS prevention and control department, and from diseases considered as opportunistic infections, cervical cancer, in my opinion, is well integrated just like tuberculosis,” (Health official, 44 years).

#### Barriers at the policy level

Barriers identified at this level included a lack of funding, a high staff turnover rate, weak supply chains, machine breakdown, and failed HPV DNA testing pilots. Among these, lack of dedicated funding and weak supply chains were the most commonly reported by health officials. These systemic challenges undermined service continuity and community confidence.

“Despite some support from external partners at the zonal level, for example, there is no dedicated budget for the screening service,” (Health official, 46 years). This view was echoed by another stakeholder: “We have failed as a country on HPV DNA testing. We had pilot sites where we attempted to establish HPV DNA testing; however, machine breakdown and reagent unavailability resulted in delayed test results,” (Health official, 44 years).

Notably, what was observed from regional and zonal officials as the main challenges to screening scale-up in the region was quite different from what is on the ground. While integration of cervical cancer screening with ART clinics and external partnerships were mentioned as a key achievement, consistent service delivery remained the main challenge according to service providers and community leaders. This misalignment suggests that policy-level progress does not necessarily translate into reliable service experience, thereby limiting its impact on screening uptake.

## Discussion

In this section, we first restate our central argument that barriers and facilitators operate hierarchically across SEM levels and then walk through each level to show how upstream failures undermine downstream facilitators, and conclude with implications for multi-level implementation strategies.

Consistent with the SEM, our findings indicate that barriers and facilitators to cervical cancer screening in South Ethiopia are distributed across all five levels of the model, but more importantly, these levels do not operate in isolation. To be explicit: Instead, we found that failures originating at the policy and institutional levels systematically undermined facilitators at the individual, interpersonal, and community levels, creating a cascade of missed opportunities that explains persistently low screening coverage despite years of national efforts. This study illustrates how barriers at the institutional and policy levels were the most influential as they undermine individual and community-level facilitators for cervical cancer screening uptake in South Ethiopia. Within this framework, determinants were not independent but hierarchically organized, with upstream system constraints shaping downstream behavioral and social processes. To clarify our line of reasoning: Our findings demonstrate a hierarchical relationship among SEM levels: when the health system fails to deliver reliable services (organizational level), women's fear of screening (individual level) is validated rather than alleviated, and community mobilization efforts (community level) lose credibility. This explains why single-domain interventions have repeatedly fallen short.

We begin our stepwise analysis at the individual level, showing how health system performance conditions personal motivation. At the individual level, both barriers and facilitators were strongly conditioned by health system performance. The strongest facilitator identified at the individual level was the threat of cervical cancer, which comes from the death of one's sister, which replaces the abstract idea of risk with something, immediate and personal. What made this facilitator powerful was not the knowledge alone, but how it interacted with the organizational level: women who had witnessed a family member's illness were willing to overcome logistical barriers only when the health system provided a clear, respectful pathway to screening. In other words, personal motivation could bridge some gaps, but not when the health system presented multiple, repeated obstacles. Our findings are consistent with a previous study^22^ and the idea of “availability heuristic” in behavioral economics,^23^ where people measure risk based on immediate examples that come to their mind. Another facilitator is the clear and empathetic counseling delivered by healthcare providers. Here again, we observed a cross-level effect: skilled provider counseling (organizational level) worked by directly modifying individual-level barriers; reducing fear, building self-efficacy, and transforming abstract risk into actionable knowledge. This finding is in line with the patient-centered model,^24^ which illustrates an increased self-efficacy and a decreased fear if patients are clearly told the “why” behind the procedure.

Conversely, chief among the identified barriers at the individual level was a profound health literacy gap. However, simply providing health education is unlikely to succeed in isolation, as fear and fatalism are reinforced by experiences at higher levels, particularly organizational unreliability. Most women residing in the region had not heard about cervical cancer, let alone understood the importance and existence of screening. Health literacy encompasses not only basic awareness but also the ability to access, understand, and act on health information.^25^ In this setting, the gap extends beyond mere knowledge to include limited perceived susceptibility and low self-efficacy for preventive action. The significant health literacy gap results in a population left behind the starting line of cervical cancer elimination. This finding is consistent with a previous study in sub-Saharan Africa,^26^ where cervical cancer is often termed a “silent killer” due to the profound lack of awareness in the region. Consistent with the findings of previous studies,^15^^,^^27^ another barrier identified is the absence of screening behavior unless clear symptoms are present. No matter how convenient the screening service is or how well-explained the service might be, women who think themselves healthy and fine never go to the screening. This symptom-based screening behavior (individual-level) becomes particularly problematic when combined with organizational barriers such as long waiting times; women who feel healthy are unwilling to invest hours at a clinic. Addressing this requires well-organized and detailed health education aimed at maximizing the perceived susceptibility of all women.^28^

Moving to the interpersonal level, we next show how social support and resistance are shaped by both community norms and health system reliability. At the interpersonal level, social support and resistance were shaped by both community norms and the reliability of health services. At this level, our findings revealed the role of husbands, peers, and health professionals in affecting women's decisions either positively or negatively. Interpersonal influences rarely stood alone. For example, a supportive husband could encourage screening, but his encouragement mattered only when the health system followed through; when the clinic had supplies, when the wait was reasonable, when treatment was available. Husbands' resistance grew when cervical cancer was seen by the community as a deadly or embarrassing illness. Community and organizational situations consistently shaped interpersonal barriers and facilitators. Supportive husbands can play an integral part in their wives' health. This finding surpasses the common focus on “seeking permission” and agrees with newly emerging strategies of engaging male partners in women's health to create a shared family asset and responsibility.^29^ Therefore, planning cervical cancer intervention strategies should involve couples rather than just women.^30^ Notably, an important cross–level interaction emerged between the interpersonal and community levels: peer experience sharing (interpersonal facilitator) was often initiated through community health worker networks (community level), suggesting that community-based mobilization can amplify interpersonal support. Simultaneously, the social network continued to be a powerful barrier. Some women remained home instead of going for screenings because of the fear of divorce in case they were found to have cervical cancer. This fear (interpersonal) was not purely social; it was reinforced by the absence of policy-level protections for women's health and by organizational failures in providing private, respectful counseling. This finding is consistent with prior literature conducted in Kenya,^31^ where women experienced a lack of permission, isolation, and abandonment. The Women's Development Army, an Ethiopian community-based health network, can also offer private counseling and escort services. Women who are unable to rely on partner support for screening services may benefit from community health worker-led outreach programs and female-only screening days.

Next, we examine the organizational level, the most immediately modifiable yet most frequently cited barrier site, and demonstrate how it reflects upstream policy constraints. At the organizational level, both facilitators and barriers reflected upstream policy and resource constraints. This level emerged as the most immediately modifiable level, yet also the site of the most frequently cited barriers. In line with the literature,^32^^,^^33^ the integration of cervical cancer screening with family planning and ART clinics was identified to be the main facilitator at the institution level. This facilitator demonstrates a positive cross–level interaction: organizational integration (FP/ART clinics) reduced individual-level barriers (travel costs, time) and increased interpersonal discussion among women attending the same clinics. This finding underscores the importance of cervical cancer screening integration into other existing units to increase the uptake. The barriers, in contrast, were a shortage of basic supplies and non-functional treatment machines, similar to the findings of a previous study.^11^ Crucially, a policy-level barrier (lack of sustainable funding for CO2 cylinder refills) manifested directly as an organizational barrier (machine breakdowns), which then became an individual-level barrier (women losing trust in the health system). The effective implementation of the “see-and-treat” cervical cancer screening approach, which Ethiopia currently implements,^3^ is significantly affected by the scarcity of consumables and the breakdown of cryotherapy machines. Therefore, while regional and zonal officials should advocate for and manage screening resources efficiently, sustainable solutions require national-level commitment.^6^

We then turn to the community level, where beliefs and stigma operate both as independent influences and as reflections of broader system weaknesses. At the community level, beliefs and stigma functioned both as independent influences and as reflections of broader system-level weaknesses. At this level, women and the entire community are more likely to receive counseling and recommendations from spiritual fathers and community elders, believing that they would not recommend something harmful to them. Community-level stigma was internalized as an individual-level barrier (shame), illustrating another cross–level interaction. This finding is consistent with the results of previous studies that explored the positive impact of elders and religious leaders in the implementation of cervical cancer research and community-based health education.^34^ On the other hand, the community can sometimes also serve as a potential barrier. Community-level barriers, specifically misinformation and religious beliefs discouraging screening, effectively nullified the facilitators observed at the interpersonal level (supportive husbands). Consistent with literature,^15^ one of the strongest barriers at this level was religious beliefs that hindered the screening acceptance of the women. The utilization of modern medical services by women is repeatedly discouraged by some religious institutions and religious teachers. To improve the uptake of cervical cancer screening and its acceptability, a community-based awareness creation campaign engaging religious leaders as allies in health is important.^26^

Finally, we ascend to the policy level, where we trace the origin of cascading failures and argue that upstream deficits represent the highest priority for sustainable change. The cascading failures at the lower levels of the SEM can be traced to profound policy-level deficits where systemic weaknesses, including lack of dedicated funding, high staff turnover, weak supply chains, non-functional equipment, and failed HPV DNA testing pilots, undermined the sustainability of screening services. To effectively scale up the uptake of cervical cancer screening, there should be a more cautious, phased approach to technology scale-up, with sustainable long-term support, rather than donor-driven pilot projects that are destined to collapse.^35^ Critically, we found that donor-dependent funding (a policy-level barrier) created an unpredictable supply chain, resulting in machine breakdowns (an organizational barrier), which in turn reduced women's trust and willingness to return for follow-up (an individual-level barrier). This cascading failure explains why single-level interventions have repeatedly failed in this setting.

This study's strengths include its triangulation of data through KIIs, IDIs, and FGDs, multi-stakeholder engagement, inclusion of four distinct Zones with different cultural characteristics, the use of SEM as a guiding framework, the diversified background of the research team, and presenting the preliminary findings of the current study at an international scientific conference. However, social desirability bias may have an impact on the results. Although the entire research team was involved in continuous reflexivity to minimize potential bias, there may still be a risk of bias. Difficulty in recruiting lost-to-follow-up women may have influenced the results. Additionally, all approached participants agreed to take part (100% agreement rate), which may indicate selection bias, as health extension workers may have referred only women likely to agree to participate. Moreover, participants did not validate final themes, and transcripts were not returned for comment.

To summarize our argument: In conclusion, a multi-level strategy that addresses the issues at every level of the SEM is necessary for effective scale-up and elimination methods. Above all, improving the deteriorating health system necessitates a steady flow of funding, equipment, and political support. To close the gaps and eliminate stigma, it is also crucial to involve the women's development army and community gatekeepers. Sustainable funding, personnel stability, and system preparedness for new screening techniques should be given top priority in policy. Future implementation science studies are needed to increase the uptake of cervical cancer screening. This includes the introduction of HPV-based screening, the implementation of community-based screening using HPV self-sampling, and the integration of cervical cancer screening into primary health care units. Moreover, the effectiveness evaluations of multi-level interventions aimed at tackling barriers from the individual to the policy level, as well as cost-effectiveness analysis comparing the integrated screening model with the vertical screening, should be conducted to inform resource allocation. Similar qualitative studies should be done in other regions of Ethiopia to explore contextual factors that facilitate or hinder the effective scale-up of cervical cancer screening.

Importantly, the significance of barriers was not equal across SEM levels. While individual and interpersonal barriers were prominent, policy-level deficits emerged as the most significant, as they produced cascading effects on institutional, community, and individual levels. Weak supply chains, lack of dedicated funding, and failed HPV DNA testing pilots undermined sustainability at all lower levels. Thus, although multi-level interventions are necessary, policy-level reforms represent the highest priority for sustainable cervical cancer screening in Ethiopia.

## Contributors

BKL: Led participant recruitment and interviews, transcribed and analyzed data, and drafted the initial manuscript. ATT: Provided input on study design, offered guidance on qualitative methodology, provided supervision, and reviewed and edited manuscript drafts. FHA: Contributed to research design, transcription and translation, and reviewing the manuscript. SFM: Provided overall supervision, secured human research ethics approval, critically revised the manuscript, and led the supporting research group. All authors had full access to the study data and are responsible for the data's integrity and the decision to submit the manuscript for publication. BKL and SFM accessed and verified the underlying data reported in the manuscript.

## Data sharing statement

The data from this study, which includes individual participant responses, will not be publicly available because of the sensitive nature of the topic and in line with Arba Minch University's ethical approval conditions. This is particularly relevant due to the small sample size and the potential for identifying the responses. If ethical approval is granted and a data access agreement is signed, we may provide de-identified snippets or thematic summaries for legitimate academic research upon a reasonable request to the corresponding author.

## Editor note

The Lancet Group takes a neutral position with respect to territorial claims in published maps and institutional affiliations.

## Declaration of interests

Begetayinoral Kussia Lahole and Selamawit Fisseha Mekuria report support from Lund University for travel, accommodation, and registration to present this research at the International Papillomavirus Society (IPVS) conference (October 23–26, 2025). All other authors declare no competing interests.
