# Supplementary material for: Challenges of scaling up cervical cancer screening in Ethiopia: a qualitative socio-ecological study
Source: eClinicalMedicine. 2026 Jul 9;97:104057. doi: 10.1016/j.eclinm.2026.104057 (PMC13380781; doi:10.1016/j.eclinm.2026.104057)
Supplement: Supplementary Material [file mmc1.pdf]

Supplementary Material for: Challenges of Scaling up Cervical Cancer Screening in Ethiopia: A Qualitative Socio-ecological Study

1. Supplementary Material 1: Adapted Social-Ecological Model (SEM) Conceptual Framework……..Page 1
2. Supplementary Material 2: Overview of FGD, KII, and IDI Guides .... ….Page 2
3. Supplementary Material 3: Coding Tree: Themes, Subthemes, and Representative Codes…………..Page 3

Supplementary Material 1: Adapted Social-Ecological Model (SEM) Conceptual Framework

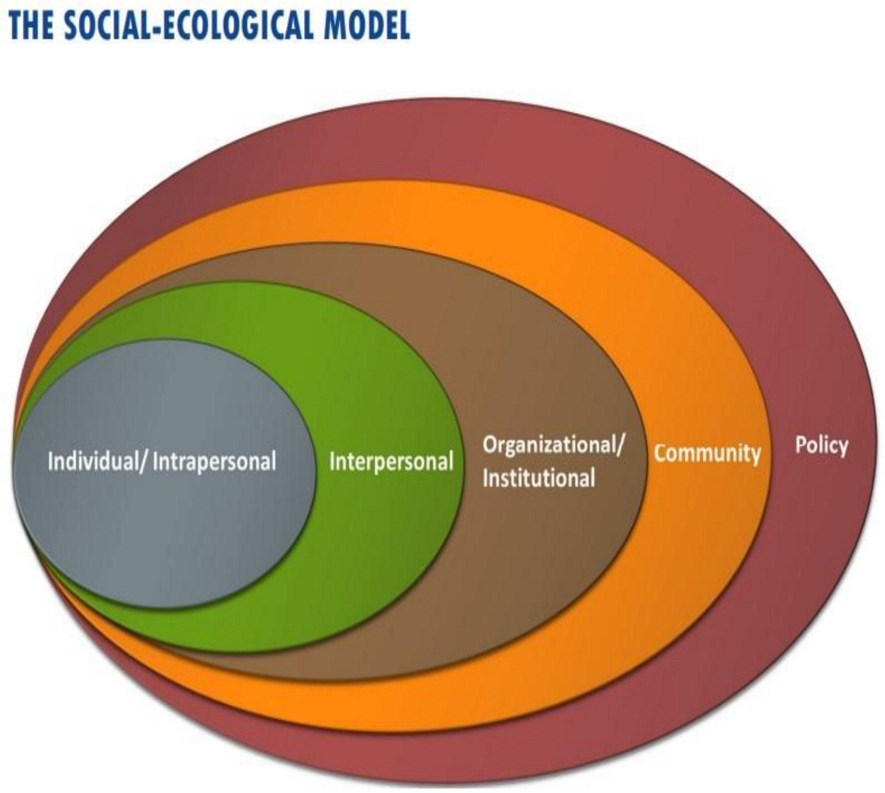

Supplementary Material 1: Adapted the social-ecological model conceptual framework for the study "Challenges of Scaling up Cervical Cancer Screening in Ethiopia: A Qualitative Socio-ecological Study."[1]

Reference

1. Desiderio G. Increasing Our Impact by Using a Social-Ecological Approach. Healthy Teen Network [Internet]. 2015 Oct 20 [cited 2026 May 3]. Available from: https://www.healthyteennetwork.org/resources/increasing-our-impact-using-social-ecological-approach/

Supplementary Material 2: Overview of Focus Group Discussion (FGD), Key Informant Interviews (KIIs), and In-Depth Interview (IDI) Guides by Participant Group

Participant Group	Method	Purpose	Key Domains Explored
Screened Women including women living with HIV (WLHIV)	FGD	Explore motivations, enablers, and experiences of cervical cancer screening	Motivators for screening; influence of HIV status WLHIV; decision influencers (health workers, peers, family); pre-screening counseling; screening experience; provider respect and support; service accessibility; recommendations
Unscreened Women	FGD	Identify barriers to first-time cervical cancer screening	Knowledge and awareness; myths, fears, and perceptions; logistical barriers (distance, cost, time, provider sex); stigma and prior healthcare experiences; social influences; preferred information and encouragement strategies
Women Diagnosed and on care	IDI	Explore drivers of adherence to follow-up care	Emotional response to diagnosis; motivators for follow-up; reminders and facility support; family and peer support; availability of follow-up services; challenges encountered; system-level improvement suggestions
Women Diagnosed but Lost to Follow-Up	IDI	Understand reasons for disengagement after diagnosis or initial treatment	Initial screening motivation; reasons for discontinuation; logistical and financial barriers; stigma, fear, and provider interactions; service navigation challenges; follow-up tracing; facilitators for re-engagement
WLHIV	IDI	Explore HIV-specific facilitators and barriers to screening and follow-up	Risk perception; Antiretroviral therapy (ART) clinic counseling; HIV-related stigma; integration of services; privacy and confidentiality; continuity of care; strategies to improve uptake and completion
Health Care Providers	KII	Assess readiness and feasibility of service integration	Roles and responsibilities; current screening practices; provider awareness; feasibility of integration; facilitators; workload and resource constraints; training and system needs
Laboratory Technologists / Pathologists	KII	Examine diagnostic capacity and its effect on care continuity	Sample processing roles; workload; availability of supplies; turnaround time; result communication; impact on uptake and follow-up; efficiency improvement strategies
Zonal & Regional Health Bureau Officials	KII	Examine policy, governance, and system-level implementation	Program oversight; uptake strategies; equity gaps; follow-up tracking; HIV integration; service capacity; policy and financing challenges
Community Leaders	IDI	Explore community perceptions and sociocultural influences	Awareness and beliefs; stigma; cultural and religious influences; barriers to access; community engagement strategies; confidentiality concerns; community-based solutions
Husbands / Male Partners	IDI	Understand male involvement in women's screening and care	Knowledge and perceptions; attitudes toward screening and treatment; support roles; cultural norms; decision-making influence; recommendations for male engagement

Supplementary Material 3: Coding Tree – Themes, Subthemes, and Representative Codes

No	Themes	Sub-themes	Included Codes
1	Health System & Structural Factors	Access &Service Quality	▪ Inconvenient Service Hours<br>▪ Lack of advanced diagnostic services<br>▪ Service Unavailability<br>▪ Shortage of trained healthcare providers<br>▪ Transportation and cost logistics<br>▪ Long Waits & Queues<br>▪ Confidentiality not kept<br>▪ Provider-patient interaction<br>▪ Providers Strong counseling
2	Interpersonal & Socio-Cultural Factors	Interpersonal & Socio-Cultural Barriers	▪ Cultural norms<br>▪ Husband's Influence<br>▪ Stigma, Shame & Privacy
		Interpersonal & Social Support Facilitators	▪ Peer experience sharing<br>▪ Religious Leader support<br>▪ Support from husband
3	Individual-Level Factors	Individual-Level Barriers	▪ Fear of procedure<br>▪ Lack of awareness<br>▪ Fear of diagnosis<br>▪ Symptom-based health-seeking behavior
		Individual-Level Facilitators	▪ Believing in Faith and Medicine<br>▪ Knowledge from Negative Example<br>▪ Personal Encounters with the Disease
4	Program & Implementation Factors	Resource & Programmatic Barriers	▪ Equipment breakdown<br>▪ High staff turnover<br>▪ Lack of dedicated funding<br>▪ Lack of essential supplies
		Enabling Program & Community Strategies	▪ Collaboration and training<br>▪ Community mobilization
5	Knowledge & Awareness	Sources of Information	▪ Information Source: Health System<br>▪ Information Source: Media<br>▪ Information Source: Peer<br>▪ Knowledge from Negative Example<br>▪ Personal Encounters with the Disease
		Awareness & Education Gaps	▪ Lack of cervical cancer education<br>▪ Not knowing service is free
